# Glucagon‐like peptide‐1 receptor agonists to expand the healthy lifespan: Current and future potentials

**DOI:** 10.1111/acel.13818

**Published:** 2023-03-20

**Authors:** Frederik Flindt Kreiner, Bernt Johan von Scholten, Peter Kurtzhals, Stephen Charles Langford Gough

**Affiliations:** ^1^ Global Medical Affairs Novo Nordisk A/S Søborg Denmark

**Keywords:** Alzheimer's disease, cardiovascular diseases, chronic kidney diseases, diabetes mellitus, glucagon‐like peptide‐1, healthy aging, non‐alcoholic steatohepatitis, obesity

## Abstract

To help ensure an expanded healthy lifespan for as many people as possible worldwide, there is a need to prevent or manage a number of prevalent chronic diseases directly and indirectly closely related to aging, including diabetes and obesity. Glucagon‐like peptide 1 receptor agonists (GLP‐1 RAs) have proven beneficial in type 2 diabetes, are amongst the few medicines approved for weight management, and are also licensed for focused cardiovascular risk reduction. In addition, strong evidence suggests several other beneficial effects of the pleiotropic peptide hormone, including anti‐inflammation. Consequently, GLP‐1 RAs are now in advanced clinical development for the treatment of chronic kidney disease, broader cardiovascular risk reduction, metabolic liver disease and Alzheimer's disease. In sum, GLP‐1 RAs are positioned as one of the pharmacotherapeutic options that can contribute to addressing the high unmet medical need characterising several prevalent aging‐related diseases, potentially helping more people enjoy a prolonged healthy lifespan.

## Graphical abstract

Alongside improved non‐pharmaceutical interventions such as lifestyle modifications and other advances, medicines have over the last decades contributed to an increasing human life expectancy. Alas, aging‐related diseases such as diabetes, obesity, cardiovascular disease and neurological disorders render the hope of a healthy lifespan elusive for many. Glucagon‐like peptide‐1 (GLP‐1) receptor agonists are amongst newer‐generation medicines that provide benefits, potentially helping more people enjoy a prolonged healthspan. Text in italics indicates potential benefits.

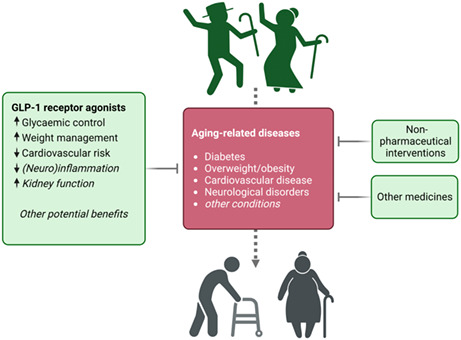

**Abbreviations**
CHIP: clonal haematopoiesis of indeterminate potential
CKD: chronic kidney disease
CVD: cardiovascular disease
CVOT: cardiovascular outcomes trial
DKD: diabetic kidney disease
DM: diabetes mellitus
GIP: gastric inhibitory peptide
GLP‐1: glucagon‐like peptide‐1
GLP‐1R: glucagon‐like peptide‐1 receptor
HFpEF: heart failure with preserved ejection fraction
MACE: major adverse cardiovascular event
NAFLD: non‐alcoholic fatty liver disease
NASH: non‐alcoholic hepatosteatosis
RA: receptor agonist
T2D: type 2 diabetes

## 1 INTRODUCTION

Increased age is associated with frailty and diseases of varying severities, and for many, the hope of a long and healthy lifespan therefore becomes elusive. Nevertheless, overall life expectancy has increased markedly during the past decades, owing to a large extent to the introduction of medicines such as statins and anti‐hypertensives. These and newer‐generation drugs have resulted in a lower prevalence and severity of age‐related illnesses such as cardiovascular disease (CVD). To sustain and reinforce this positive trend and help ensure a prolonged healthspan for more people across the world, novel pharmacotherapeutics and optimal use of existing options are arguably needed.

Glucagon‐like peptide‐1 (GLP‐1) receptor agonists (RAs) are an example of a drug class with proven or potential benefits across a range of prevalent age‐related conditions and complications (Müller et al., 2019). Originally developed to manage blood glucose levels in type 2 diabetes (T2D), GLP‐1 RAs have subsequently been confirmed to have marked benefits on body weight and CVD risk. Furthermore, evidence from research and clinical use of the drug class has led to the initiation of clinical trials with GLP‐1 RAs in other prominent aging‐related diseases, including chronic kidney disease (CKD) and Alzheimer's disease.

## 2 THE PLEIOTROPIC GLP‐1 HORMONE AND DRUG CLASS

**FIGURE 1:**
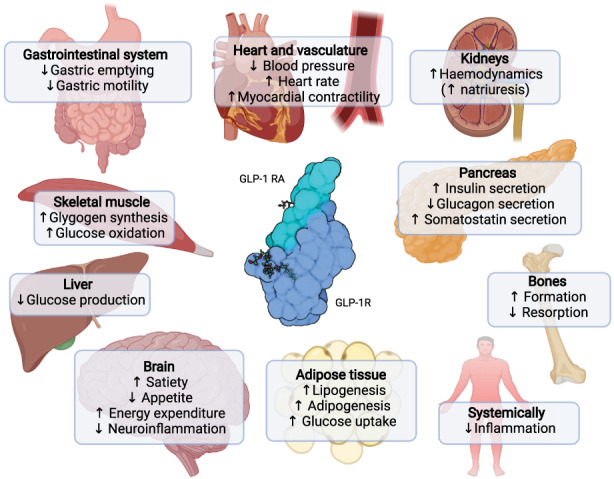
Biological actions and indirect effects of glucagon‐like peptide 1. Major direct and indirect effects of glucagon‐like peptide‐1 (GLP‐1) in humans across organ systems. Both confirmed and potential effects are shown. The GLP‐1 receptor agonist (RA) semaglutide is shown bound to the GLP‐1 receptor (GLP‐1R). Created with BioRender.com.

Secreted into systemic circulation following food intake, GLP‐1 is one of two major gut‐derived peptide hormones of the incretin system (the other is gastric inhibitory peptide, GIP) and plays important roles in sustaining glucose homeostasis via its glucose‐dependent insulinotropic and glucagonostatic effects (Müller et al., 2019). GLP‐1 is a pleiotropic hormone that exerts its actions via the GLP‐1 receptor (GLP‐1R), which in humans has been shown to be expressed in the pancreas and several other tissues, including many areas of the brain (Figure 1).

Pharmaceutical development has enabled long‐acting GLP‐1 RAs for once‐daily and once‐weekly subcutaneous injection (Müller et al., 2019; Nauck et al., 2020). Recently, a GLP‐1 RA (semaglutide) was introduced for oral administration as a tablet. GLP‐1 RAs have proven safe and well tolerated during development and in clinical use according to the prescribing information for each of the marketed GLP‐1 RAs. Tolerability concerns related mainly to gastrointestinal side effects can be mitigated for most using dose‐escalation regimens. Detailed reviews of the safety and tolerability profiles of GLP‐1 RAs are available elsewhere (Amaro et al., 2022; Trujillo, 2020) and in the reports cited below for each of the aging‐related conditions discussed in this article.

GLP‐1 RAs are currently indicated to improve glycaemic control in T2D, for weight management, and to reduce CVD risk in people with T2D.

T2D and obesity are highly and increasingly prevalent cardiometabolic diseases that may accelerate the aging process, illustrating the need for timely anti‐diabetes and anti‐obesity intervention to help prevent or delay age‐associated complications such as CVD, kidney disease and neurodegenerative disorders (Morley, 2008; Salvestrini et al., 2019).

### 2.1 Type 2 diabetes

Amongst people 65 years of age or older, the T2D prevalence is high in many societies and in the US specifically, 25% of the people in this age group is estimated to have diabetes with even more having prediabetes (Rooney et al., 2021). Furthermore, older adults are amongst those with the fastest growing diabetes incidence rate (Bellary et al., 2021; Khan et al., 2020) and those with the highest prevalence of diabetes‐related comorbidities and poor outcomes (Bellary et al., 2021; Laiteerapong et al., 2011; Quiñones et al., 2019). The metabolic derangements in T2D are caused or exacerbated further by the physiological changes that occur with aging, including reduced physical functioning and sarcopenia (Bellary et al., 2021). Overall, GLP‐1 RAs in T2D provide meaningful improvements in glycaemic control regardless of age to allow recipients to achieve the glycaemia‐focused treatment goals (Nauck et al., 2020). Compared with other glucose‐lowering drugs, GLP‐1 RAs ‐ because of their glucose‐dependent mode of action ‐ are associated with low risk of hypoglycaemia, an important consideration in older persons with T2D.

### 2.2 Weight management

Physiologically, humans tend to gain excess body weight when aging, but overweight and in particular obesity negatively impacts healthspan and markedly decreases quality of life, with visceral and ectopic adiposity being especially associated with cardiometabolic disease and premature death. Recently, the European Commission classified obesity as a chronic disease and regardless of age, a weight loss of 5% has traditionally been regarded as clinically relevant, with 10% now emerging as the minimum to substantially lower the risk of poor outcomes (Tahrani & Morton, 2022).

Liraglutide, the first GLP‐1 RA licensed for use in weight management, was approved in 2013 and recently, a second‐generation RA, semaglutide, was approved with weight loss of up to around 18% after 68 weeks shown in phase 3 development (Wadden et al., 2021). In addition, tirzepatide, a GLP‐1/GIP dual RA, appears promising in obesity management with weight loss of up to around 21% after 72 weeks in phase 3 (Jastreboff et al., 2022). Further, a product combining semaglutide with an amylin analogue is in phase 3 development (Table 1), showing weight loss of up to 17% after only 20 weeks in a phase 1 trial (Enebo et al., 2021). Current knowledge links weight loss with GLP‐1 RAs to central effects in brain regions expressing the GLP‐1R, modifying food preferences, reducing appetite and food intake, and increasing satiety. The weight‐reducing effects of GLP‐1 RAs (liraglutide and semaglutide) do not depend on age (Novo Nordisk A/S, 2015, 2022).

**TABLE 1:** Major clinical trials with GLP‐1 RAs in aging‐related diseases.

Trial name	Title	Clinicaltrials.gov ID
Weight management		
OASIS 1	Effects of oral semaglutide on body weight in overweight/obesity	NCT05035095
REDEFINE 2	Effects of semaglutide + cagrilintide (amylin analogue) in overweight/obesity	NCT05394519
Cardiovascular risk reduction		
SELECT	Effects of semaglutide on heart disease and stroke in overweight/obesity	NCT03574597
STEP‐HFpEF	Effects of semaglutide on HFpEF in overweight/obesity	NCT04788511
STEP‐HFpEF DM	Effects of semaglutide on HFpEF in overweight/obesity with type 2 diabetes	NCT04916470
STRIDE	Effects of semaglutide in T2D with peripheral arterial disease	NCT04560998
SURPASS‐CVOT	Effects of tirzepatide on MACE in type 2 diabetes	NCT04255433
Chronic kidney disease		
FLOW	Effects of semaglutide on kidney outcomes in type 2 diabetes with chronic kidney disease	NCT03819153
REMODEL	Mechanisms of action of semaglutide in type 2 diabetes with chronic kidney disease	NCT04865770
Non‐alcoholic steatohepatitis		
ESSENCE	Effect of semaglutide in non‐alcoholic steatohepatitis	NCT04822181
REALIST	Effect of dulaglutide in non‐alcoholic steatohepatitis	NCT03648554
Neurological disorders		
EVOKE and EVOKE+	Effects of semaglutide in early Alzheimer's disease	NCT04777396 NCT04777409
Exenatide‐PD3	Exenatide as disease modifying treatment in Parkinson's disease	NCT04232969
GAPP‐SVD	Exenatide in preventing progression of small vessel disease	NCT05356104

*Note*: List is not exhaustive and includes representative major, late‐stage clinical trials in aging‐related diseases.

Abbreviations: CVOT, cardiovascular outcomes trial; DM, diabetes mellitus; HFpEF, heart failure with preserved ejection fraction; MACE, major adverse cardiovascular event.

### 2.3 Cardiovascular disease

CVD remains the major cause of morbidity and mortality globally, and the incidence and prevalence of CVD depend on age. The predominant cause of CVD is atherosclerosis, representing a decades‐long process prior to clinical manifestation.

Strong evidence of a cardiovascular risk reduction benefit of GLP‐1 RAs in people with T2D has accumulated with data from large cardiovascular outcomes trials (CVOT) collectively showing a significant relative risk reduction of 14% for the 3‐component MACE composite outcome (major cardiovascular adverse events, comprising cardiovascular death, myocardial infarction, and non‐fatal stroke) (Sattar et al., 2021). Moreover, significant 11%–12% relative risk reductions for all‐cause mortality and for heart failure hospitalisations have also been shown (Sattar et al., 2021).

Currently, dulaglutide, liraglutide and semaglutide are approved for cardiovascular risk reduction in people with T2D with established CVD or increased cardiovascular risk. Additional CVD‐focused trials in T2D and weight management are currently ongoing (Table 1).

The cardiovascular benefits of GLP‐1 RAs appear to be driven by both direct and indirect effects (Figure 1); as such, the cardiac effects of GLP‐1 RAs are partly independent of their effects on body weight and may also at least partly be mediated via direct effects via the GLP‐1R (Müller et al., 2019). Other evidence points towards that the cardiovascular benefits may be driven by reduction of visceral fat (Neeland et al., 2021). Studies in animal models of atherosclerosis have demonstrated anti‐inflammatory and anti‐atherosclerotic effects of GLP‐1 RAs (Rakipovski et al., 2018). GLP‐1 RAs can lower blood triglyceride levels (Patel et al., 2014), which may also add to an anti‐atherosclerotic effect.

## 3 FUTURE POTENTIALS AND PERSPECTIVES

### 3.1 Chronic kidney disease

CKD contributes markedly to the increased morbidity or mortality of the elderly. CKD in people with diabetes (diabetic kidney disease, DKD) can, at least partly, be seen as the renal manifestation of the diabetes‐related glucotoxicity (Thomas et al., 2015). Other risk factors include dyslipidaemia, obesity, and hypertension. CKD/DKD and CVD are strongly linked; DKD in particular is associated with an unfavourable cardiovascular prognosis. Accordingly, the CVOTs mentioned earlier also suggested a kidney‐protective benefit of GLP‐1 RAs, indicating significant relative risk reductions of up to 35% on composite kidney outcomes with real‐world evidence corroborating this potential (Sattar et al., 2021; von Scholten et al., 2022). Currently, completion of ongoing kidney‐specific outcomes trials (Table 1) and regulatory approval are needed for wide‐spread use of GLP‐1 RAs in CKD. Potential direct kidney‐beneficial effects of GLP‐1 RAs include improved haemodynamics (via RAAS modulation and increased natriuresis), anti‐inflammation and reduced oxidative stress (von Scholten et al., 2022). Indirect effects on blood pressure and body weight may also play a role.

### 3.2 Alzheimer's disease and other neurological disorders

Alzheimer's disease is one of the most common neurodegenerative disorders, the risk of which increases with age. Based on evidence from clinical trials and preclinical studies and in line with the fact that Alzheimer's disease is intimately associated with diabetes to the extent that some view the disease as “type 3 diabetes” (Kandimalla et al., 2017), GLP‐1 RAs are being repurposed as a potential intervention to address the unmet medical need in Alzheimer's disease. Preclinical data have indicated a neuroprotective role for GLP‐1, and current evidence points towards reduced inflammation as the common denominator for the benefits in Alzheimer's disease and other neurological disorders (Cai et al., 2018; Li et al., 2021; Nørgaard et al., 2022; Yun et al., 2018; Zhao et al., 2020). Post hoc analysis of datasets from CVOTs in T2D have indicated a significantly 53% lower risk of dementia for people treated with GLP‐1 RAs liraglutide or semaglutide (Nørgaard et al., 2022), and an exploratory analysis of data from the REWIND CVOT indicated a robust 14% reduction of risk of substantive cognitive impairment with dulaglutide (Cukierman‐Yaffe et al., 2020). Currently, the effects of semaglutide (oral administration) in Alzheimer's disease are being investigated in phase 3 trials (Table 1).

In addition to Alzheimer's disease, a potential for GLP‐1 RAs has also been shown in neurological disorders such as Parkinson's disease (Athauda et al., 2017; Brauer et al., 2020) and cerebral small vessel disease (Zhao et al., 2020) with ongoing clinical studies exploring the possibilities (Table 1).

### 3.3 Metabolic liver disease

Amongst the non‐alcoholic fatty liver diseases (NAFLDs), non‐alcoholic steatohepatitis (NASH) is one of the most common, associated with serious complications such as cirrhosis, CVD and CKD, but without effective pharmacotherapeutic options (Younossi et al., 2018). NASH is closely linked to diabetes and obesity; for example, estimates have shown that amongst people with T2D, around two‐thirds have NASH (Younossi et al., 2018, 2019). In addition, although NASH can occur at any age, advanced age is a major risk factor. A meta‐analysis showed that GLP‐1 RAs may resolve NAFLD (Ghosal et al., 2021) and recently, a phase 2 study showed that the GLP‐1 RA semaglutide was able to provide significant resolution of NASH (Newsome et al., 2021). Additional clinical trials are ongoing to establish the potential clinical usefulness of GLP‐1 RAs in NASH (Table 1).

### 3.4 Anti‐inflammation

Metabolic diseases such as diabetes and obesity in themselves confer an increased inflammatory burden, which plays pathogenic roles in several complications. In addition, mechanisms such as clonal haematopoiesis of indeterminate potential (CHIP) (Jaiswal & Libby, 2020) appear to be driving inflammatory processes associated with aging, including introduction of proinflammatory mutations in immune cells. Several preclinical and clinical investigations have shown that GLP‐1 RA treatment is anti‐inflammatory (Zobel et al., 2021), which in addition to the disease‐specific benefits mentioned above may be especially helpful in reducing the overall inflammatory burden associated with aging as the result of CHIP and/or other processes.

## 4 SUMMARY

In sum, GLP‐1 RAs provide proven and potential benefits that may help people experience a prolonged healthy lifespan with reduced risk of serious and chronic aging‐related conditions. Thus, the drug class is positioned as a novel pharmacotherapeutic option that in combination with non‐pharmaceutical interventions can help address the pronounced medical need associated with the aging human population.

## AUTHORS CONTRIBUTIONS

All authors contributed equally to the preparation of the article.

## CONFLICT OF INTEREST STATEMENT

All authors are employees and shareholders of Novo Nordisk A/S.

## Data Availability

Data sharing is not applicable to this article as no new data were created or analyzed in this study.

## References

[acel13818-bib-0001] Amaro, A. , Sugimoto, D. , & Wharton, S. (2022). Efficacy and safety of semaglutide for weight management: Evidence from the STEP program. Postgraduate Medicine, 134(sup1), 5–17. 10.1080/00325481.2022.2147326 36691309

[acel13818-bib-0002] Athauda, D. , Maclagan, K. , Skene, S. S. , Bajwa‐Joseph, M. , Letchford, D. , Chowdhury, K. , Hibbert, S. , Budnik, N. , Zampedri, L. , Dickson, J. , Li, Y. , Aviles‐Olmos, I. , Warner, T. T. , Limousin, P. , Lees, A. J. , Greig, N. H. , Tebbs, S. , & Foltynie, T. (2017). Exenatide once weekly versus placebo in Parkinson's disease: A randomised, double‐blind, placebo‐controlled trial. Lancet, 390(10103), 1664–1675. 10.1016/s0140-6736(17)31585-4 28781108 PMC5831666

[acel13818-bib-0003] Bellary, S. , Kyrou, I. , Brown, J. E. , & Bailey, C. J. (2021). Type 2 diabetes mellitus in older adults: Clinical considerations and management. Nature Reviews. Endocrinology, 17(9), 534–548. 10.1038/s41574-021-00512-2 34172940

[acel13818-bib-0004] Brauer, R. , Wei, L. , Ma, T. , Athauda, D. , Girges, C. , Vijiaratnam, N. , Auld, G. , Whittlesea, C. , Wong, I. , & Foltynie, T. (2020). Diabetes medications and risk of Parkinson's disease: A cohort study of patients with diabetes. Brain, 143(10), 3067–3076. 10.1093/brain/awaa262 33011770 PMC7794498

[acel13818-bib-0005] Cai, H. Y. , Yang, J. T. , Wang, Z. J. , Zhang, J. , Yang, W. , Wu, M. N. , & Qi, J. S. (2018). Lixisenatide reduces amyloid plaques, neurofibrillary tangles and neuroinflammation in an APP/PS1/tau mouse model of Alzheimer's disease. Biochemical and Biophysical Research Communications, 495(1), 1034–1040. 10.1016/j.bbrc.2017.11.114 29175324

[acel13818-bib-0006] Cukierman‐Yaffe, T. , Gerstein, H. C. , Colhoun, H. M. , Diaz, R. , García‐Pérez, L. E. , Lakshmanan, M. , Bethel, A. , Xavier, D. , Probstfield, J. , Riddle, M. C. , Rydén, L. , Atisso, C. M. , Hall, S. , Rao‐Melacini, P. , Basile, J. , Cushman, W. C. , Franek, E. , Keltai, M. , Lanas, F. , … Temelkova‐Kurktschiev, T. (2020). Effect of dulaglutide on cognitive impairment in type 2 diabetes: An exploratory analysis of the REWIND trial. Lancet Neurology, 19(7), 582–590. 10.1016/S1474-4422(20)30173-3 32562683

[acel13818-bib-0007] Enebo, L. B. , Berthelsen, K. K. , Kankam, M. , Lund, M. T. , Rubino, D. M. , Satylganova, A. , & Lau, D. C. W. (2021). Safety, tolerability, pharmacokinetics, and pharmacodynamics of concomitant administration of multiple doses of cagrilintide with semaglutide 2.4 mg for weight management: A randomised, controlled, phase 1b trial. Lancet, 397(10286), 1736–1748. 10.1016/S0140-6736(21)00845-X 33894838

[acel13818-bib-0008] Ghosal, S. , Datta, D. , & Sinha, B. (2021). A meta‐analysis of the effects of glucagon‐like‐peptide 1 receptor agonist (GLP1‐RA) in nonalcoholic fatty liver disease (NAFLD) with type 2 diabetes (T2D). Scientific Reports, 11(1), 22063. 10.1038/s41598-021-01663-y 34764398 PMC8586228

[acel13818-bib-0009] Jaiswal, S. , & Libby, P. (2020). Clonal haematopoiesis: Connecting ageing and inflammation in cardiovascular disease. Nature Reviews. Cardiology, 17(3), 137–144. 10.1038/s41569-019-0247-5 31406340 PMC9448847

[acel13818-bib-0010] Jastreboff, A. M. , Aronne, L. J. , Ahmad, N. N. , Wharton, S. , Connery, L. , Alves, B. , Kiyosue, A. , Zhang, S. , Liu, B. , Bunck, M. C. , Stefanski, A. , & Investigators, S . (2022). Tirzepatide once weekly for the treatment of obesity. The New England Journal of Medicine, 387(3), 205–216. 10.1056/NEJMoa2206038 35658024

[acel13818-bib-0011] Kandimalla, R. , Thirumala, V. , & Reddy, P. H. (2017). Is Alzheimer's disease a type 3 diabetes? A critical appraisal. Biochimica et Biophysica Acta – Molecular Basis of Disease, 1863(5), 1078–1089. 10.1016/j.bbadis.2016.08.018 27567931 PMC5344773

[acel13818-bib-0012] Khan, M. A. B. , Hashim, M. J. , King, J. K. , Govender, R. D. , Mustafa, H. , & Al Kaabi, J. (2020). Epidemiology of type 2 diabetes – global burden of disease and forecasted trends. Journal of Epidemiology and Global Health, 10(1), 107–111. 10.2991/jegh.k.191028.001 32175717 PMC7310804

[acel13818-bib-0013] Laiteerapong, N. , Karter, A. J. , Liu, J. Y. , Moffet, H. H. , Sudore, R. , Schillinger, D. , John, P. M. , & Huang, E. S. (2011). Correlates of quality of life in older adults with diabetes: The diabetes & aging study. Diabetes Care, 34(8), 1749–1753. 10.2337/dc10-2424 21636795 PMC3142040

[acel13818-bib-0014] Li, Z. , Chen, X. , Vong, J. S. L. , Zhao, L. , Huang, J. , Yan, L. Y. C. , Ip, B. , Wing, Y. K. , Lai, H. M. , Mok, V. C. T. , & Ko, H. (2021). Systemic GLP‐1R agonist treatment reverses mouse glial and neurovascular cell transcriptomic aging signatures in a genome‐wide manner. Communications Biology, 4(1), 656. 10.1038/s42003-021-02208-9 34079050 PMC8172568

[acel13818-bib-0015] Morley, J. E. (2008). Diabetes and aging: Epidemiologic overview. Clinics in Geriatric Medicine, 24(3), 395–405, v. 10.1016/j.cger.2008.03.005 18672179

[acel13818-bib-0016] Müller, T. D. , Finan, B. , Bloom, S. R. , D’Alessio, D. , Drucker, D. J. , Flatt, P. R. , Fritsche, A. , Gribble, F. , Grill, H. J. , Habener, J. F. , Holst, J. J. , Langhans, W. , Meier, J. J. , Nauck, M. A. , Perez‐Tilve, D. , Pocai, A. , Reimann, F. , Sandoval, D. A. , Schwartz, T. W. , … Tschöp, M. H. (2019). Glucagon‐like peptide 1 (GLP‐1). Molecular Metabolism, 30, 72–130. 10.1016/j.molmet.2019.09.010 31767182 PMC6812410

[acel13818-bib-0017] Nauck, M. A. , Quast, D. R. , Wefers, J. , & Meier, J. J. (2020). GLP‐1 receptor agonists in the treatment of type 2 diabetes – state‐of‐the‐art. Molecular Metabolism, 46, 101102. 10.1016/j.molmet.2020.101102 33068776 PMC8085572

[acel13818-bib-0018] Neeland, I. J. , Marso, S. P. , Ayers, C. R. , Lewis, B. , Oslica, R. , Francis, W. , Rodder, S. , Pandey, A. , & Joshi, P. H. (2021). Effects of liraglutide on visceral and ectopic fat in adults with overweight and obesity at high cardiovascular risk: A randomised, double‐blind, placebo‐controlled, clinical trial. The Lancet Diabetes and Endocrinology, 9(9), 595–605. 10.1016/s2213-8587(21)00179-0 34358471

[acel13818-bib-0019] Newsome, P. N. , Buchholtz, K. , Cusi, K. , Linder, M. , Okanoue, T. , Ratziu, V. , Sanyal, A. J. , Sejling, A. S. , & Harrison, S. A. (2021). A placebo‐controlled trial of subcutaneous semaglutide in nonalcoholic steatohepatitis. The New England Journal of Medicine, 384(12), 1113–1124. 10.1056/NEJMoa2028395 33185364

[acel13818-bib-0020] Nørgaard, C. H. , Friedrich, S. , Hansen, C. T. , Gerds, T. , Ballard, C. , Møller, D. V. , Knudsen, L. B. , Kvist, K. , Zinman, B. , Holm, E. , Torp‐Pedersen, C. , & Mørch, L. S. (2022). Treatment with glucagon‐like peptide‐1 receptor agonists and incidence of dementia: Data from pooled double‐blind randomized controlled trials and nationwide disease and prescription registers. Alzheimer's & Dementia, 8(1), e12268. 10.1002/trc2.12268 PMC8864443 35229024

[acel13818-bib-0021] Novo Nordisk A/S . (2015). Saxenda® (liraglutide), EU Summary of Product Characteristics (SmPC).

[acel13818-bib-0022] Novo Nordisk A/S . (2022). Wegovy® (semaglutide), EU Summary of Product Characteristics (SmPC).

[acel13818-bib-0023] Patel, V. J. , Joharapurkar, A. A. , Shah, G. B. , & Jain, M. R. (2014). Effect of GLP‐1 based therapies on diabetic dyslipidemia. Current Diabetes Reviews, 10(4), 238–250. 10.2174/1573399810666140707092506 24998439

[acel13818-bib-0024] Quiñones, A. R. , Markwardt, S. , & Botoseneanu, A. (2019). Diabetes‐multimorbidity combinations and disability among middle‐aged and older adults. Journal of General Internal Medicine, 34(6), 944–951. 10.1007/s11606-019-04896-w 30815788 PMC6544693

[acel13818-bib-0025] Rakipovski, G. , Rolin, B. , Nøhr, J. , Klewe, I. , Frederiksen, K. S. , Augustin, R. , Hecksher‐Sørensen, J. , Ingvorsen, C. , Polex‐Wolf, J. , & Knudsen, L. B. (2018). The GLP‐1 analogs liraglutide and Semaglutide reduce atherosclerosis in ApoE(−/−) and LDLr(−/−) mice by a mechanism that includes inflammatory pathways. JACC: Basic to Translational Science, 3(6), 844–857. 10.1016/j.jacbts.2018.09.004 30623143 PMC6314963

[acel13818-bib-0026] Rooney, M. R. , Rawlings, A. M. , Pankow, J. S. , Echouffo Tcheugui, J. B. , Coresh, J. , Sharrett, A. R. , & Selvin, E. (2021). Risk of progression to diabetes among older adults with prediabetes. JAMA Internal Medicine, 181(4), 511–519. 10.1001/jamainternmed.2020.8774 33555311 PMC7871207

[acel13818-bib-0027] Salvestrini, V. , Sell, C. , & Lorenzini, A. (2019). Obesity may accelerate the aging process. Frontiers in Endocrinology, 10, 266. 10.3389/fendo.2019.00266 31130916 PMC6509231

[acel13818-bib-0028] Sattar, N. , Lee, M. M. Y. , Kristensen, S. L. , Branch, K. R. H. , Del Prato, S. , Khurmi, N. S. , Lam, C. S. P. , Lopes, R. D. , McMurray, J. J. V. , Pratley, R. E. , Rosenstock, J. , & Gerstein, H. C. (2021). Cardiovascular, mortality, and kidney outcomes with GLP‐1 receptor agonists in patients with type 2 diabetes: A systematic review and meta‐analysis of randomised trials. The Lancet Diabetes and Endocrinology, 9(10), 653–662. 10.1016/S2213-8587(21)00203-5 34425083

[acel13818-bib-0029] Tahrani, A. A. , & Morton, J. (2022). Benefits of weight loss of 10% or more in patients with overweight or obesity: A review. Obesity (Silver Spring), 30(4), 802–840. 10.1002/oby.23371 35333446

[acel13818-bib-0030] Thomas, M. C. , Brownlee, M. , Susztak, K. , Sharma, K. , Jandeleit‐Dahm, K. A. , Zoungas, S. , Rossing, P. , Groop, P. H. , & Cooper, M. E. (2015). Diabetic kidney disease. Nature Reviews. Disease Primers, 1, 15018. 10.1038/nrdp.2015.18 PMC7724636 27188921

[acel13818-bib-0031] Trujillo, J. (2020). Safety and tolerability of once‐weekly GLP‐1 receptor agonists in type 2 diabetes. Journal of Clinical Pharmacy and Therapeutics, 45(Suppl 1), 43–60. 10.1111/jcpt.13225 32910487 PMC7540535

[acel13818-bib-0032] von Scholten, B. J. , Kreiner, F. F. , Rasmussen, S. , Rossing, P. , & Idorn, T. (2022). The potential of GLP‐1 receptor agonists in type 2 diabetes and chronic kidney disease: From randomised trials to clinical practice. Therapeutic Advances in Endocrinology and Metabolism, 13, 20420188221112490. 10.1177/20420188221112490 35874312 PMC9301118

[acel13818-bib-0033] Wadden, T. A. , Bailey, T. S. , Billings, L. K. , Davies, M. , Frias, J. P. , Koroleva, A. , Lingvay, I. , O’Neil, P. M. , Rubino, D. M. , Skovgaard, D. , Wallenstein, S. O. R. , Garvey, W. T. , & Investigators, S. (2021). Effect of subcutaneous Semaglutide vs placebo as an adjunct to intensive behavioral therapy on body weight in adults with overweight or obesity: The STEP 3 randomized clinical trial. Journal of the American Medical Association, 325, 1403–1413. 10.1001/jama.2021.1831 33625476 PMC7905697

[acel13818-bib-0034] Younossi, Z. , Anstee, Q. M. , Marietti, M. , Hardy, T. , Henry, L. , Eslam, M. , George, J. , & Bugianesi, E. (2018). Global burden of NAFLD and NASH: Trends, predictions, risk factors and prevention. Nature Reviews. Gastroenterology & Hepatology, 15(1), 11–20. 10.1038/nrgastro.2017.109 28930295

[acel13818-bib-0035] Younossi, Z. M. , Golabi, P. , de Avila, L. , Paik, J. M. , Srishord, M. , Fukui, N. , Qiu, Y. , Burns, L. , Afendy, A. , & Nader, F. (2019). The global epidemiology of NAFLD and NASH in patients with type 2 diabetes: A systematic review and meta‐analysis. Journal of Hepatology, 71(4), 793–801. 10.1016/j.jhep.2019.06.021 31279902

[acel13818-bib-0036] Yun, S. P. , Kam, T. I. , Panicker, N. , Kim, S. , Oh, Y. , Park, J. S. , Kwon, S. H. , Park, Y. J. , Karuppagounder, S. S. , Park, H. , Kim, S. , Oh, N. , Kim, N. A. , Lee, S. , Brahmachari, S. , Mao, X. , Lee, J. H. , Kumar, M. , An, D. , … Ko, H. S. (2018). Block of A1 astrocyte conversion by microglia is neuroprotective in models of Parkinson's disease. Nature Medicine, 24(7), 931–938. 10.1038/s41591-018-0051-5 PMC6039259 29892066

[acel13818-bib-0037] Zhao, L. , Li, Z. , Vong, J. S. L. , Chen, X. , Lai, H. M. , Yan, L. Y. C. , Huang, J. , Sy, S. K. H. , Tian, X. , Huang, Y. , Chan, H. Y. E. , So, H. C. , Ng, W. L. , Tang, Y. , Lin, W. J. , Mok, V. C. T. , & Ko, H. (2020). Pharmacologically reversible zonation‐dependent endothelial cell transcriptomic changes with neurodegenerative disease associations in the aged brain. Nature Communications, 11(1), 4413. 10.1038/s41467-020-18249-3 PMC7474063 32887883

[acel13818-bib-0038] Zobel, E. H. , Ripa, R. S. , von Scholten, B. J. , Rotbain Curovic, V. , Kjaer, A. , Hansen, T. W. , Rossing, P. , & Størling, J. (2021). Effect of liraglutide on expression of inflammatory genes in type 2 diabetes. Scientific Reports, 11(1), 18522. 10.1038/s41598-021-97967-0 34535716 PMC8448739

